# The LSmAD Domain of Ataxin-2 Modulates the Structure and RNA Binding of Its Preceding LSm Domain

**DOI:** 10.3390/cells14050383

**Published:** 2025-03-06

**Authors:** Shengping Zhang, Yunlong Zhang, Ting Chen, Hong-Yu Hu, Changrui Lu

**Affiliations:** 1 College of Biological Science and Medical Engineering, Donghua University, Shanghai 201620, China; zhangshengping2018@163.com (S.Z.); zhyl@dhu.edu.cn (Y.Z.); chenting@dhu.edu.cn (T.C.); 2 Key Laboratory of RNA Innovation, Science and Engineering, Shanghai Institute of Biochemistry and Cell Biology, Center for Excellence in Molecular Cell Science, Chinese Academy of Sciences, Shanghai 200031, China

**Keywords:** Ataxin-2, LSm, LSmAD, RNA binding, U-rich, SHAPE

## Abstract

Ataxin-2 (Atx2), an RNA-binding protein, plays a pivotal role in the regulation of RNA, intracellular metabolism, and translation within the cellular environment. Although both the Sm-like (LSm) and LSm-associated (LSmAD) domains are considered to associated with RNA binding, there is still a lack of experimental evidence supporting their functions. To address this, we designed and constructed several recombinants containing the RNA-binding domain (RBD) of Atx2. By employing biophysical and biochemical techniques, such as EMSA and SHAPE chemical detection, we identified that LSm is responsible for RNA binding, whereas LSmAD alone does not bind RNA. NMR and small-angle X-ray scattering (SAXS) analyses have revealed that the LSmAD domain exhibits limited structural integrity and poor folding capability. The EMSA data confirmed that both LSm and LSm-LSmAD bind RNA, whereas LSmAD alone cannot, suggesting that LSmAD may serve as an auxiliary role to the LSm domain. SHAPE chemical probing further demonstrates that LSm binds to the AU-rich, GU-rich, or CU-rich sequence, but not to the CA-rich sequence. These findings indicate that Atx2 can interact with the U-rich sequences in the 3′-UTR, implicating its role in poly(A) tailing and the regulation of mRNA translation and degradation.

## 1. Introduction

Sm and LSm proteins form complexes and interact with RNA across all eukaryotic organisms, participating in a broad array of cellular processes [1,2,3,4]. Eukaryotic LSm proteins and their bacterial counterparts play significant roles in RNA processing and degradation [2,5,6,7]. Ataxin-2 (Atx2) belongs to the LSm protein family [8] and contains three conserved regions: an LSm domain, an LSmAD, and a putative poly(A)-binding protein-interacting motif (PAM2) flanked by extended intrinsically disordered regions (IDRs) [9,10]. Studies in Drosophila have demonstrated that the C-terminal IDR of Atx2 participates in the assembly of mRNPs into particles [11]. The LSm domain is likely involved in RNA binding, while the LSmAD contains signals associated with clathrin-mediated anti-Golgi processes [12,13]. The Sm proteins belong to a highly conserved family of RNA-binding proteins (RBPs). RBPs regulate gene expression and play a fundamental role in maintaining cellular and organismal homeostasis. These proteins influence various aspects of RNA metabolism, including subcellular localization, translation efficiency, and degradation, by binding to target mRNA molecules and modulating the expression of the encoded proteins [14,15,16]. Atx2, an evolutionarily conserved RBP [17,18], has been identified as a genetic determinant and risk factor for several diseases, such as spinocerebellar ataxia type II (SCA2) and amyotrophic lateral sclerosis (ALS) [9,11,19,20,21,22,23,24,25,26,27,28,29,30,31]. Atx2 primarily localizes in the cytoplasm and is potentially involved in regulating RNA stability, localization, and translation [32].

**Figure 1:**
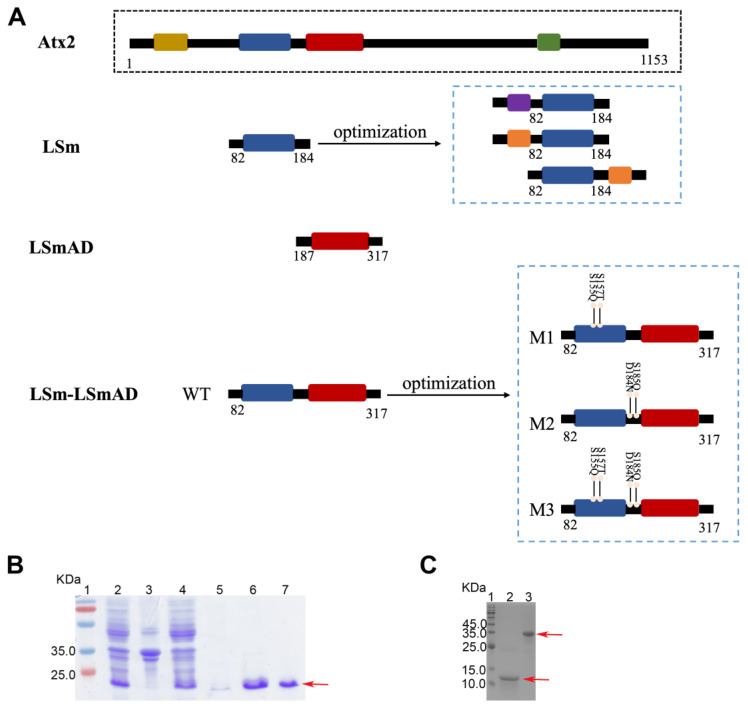
Design, expression, and purification of the recombinant domains of Atx2 for biochemical analyses. (**A**) Domain architecture of Atx2. Atx2 contains a polyQ tract at its N-terminus, the LSm and LSmAD domains, and a PAM2 domain flanked by IDRs. The polyQ, LSm, LSmAD, and PAM2 domains of Atx2 are shown in khaki, blue, red, and green, respectively. GB1 and SUMO fusion tags are represented in purple and orange, respectively. The numbers are given for the start and end residues of structural regions. (**B**) Expression and purification of LSmAD: lane 1, molecular weight marker; lane 2, cell lysates (induced); lane 3, precipitate; lane 4, supernatant; lane 5, protein sample eluted with 20 mM imidazole; lane 6, protein sample eluted with 250 mM imidazole; lane 7, purified sample by SEC-FPLC. (**C**) Preparation of LSm and the M2 mutant of LSm-LSmAD: lane 1, protein marker; lane 2, LSm; lane 3, M2. The arrow represents the target protein of interest.

The *ATXN2* gene is evolutionarily conserved across many species [14]. The yeast homolog of Ataxin-2, Pbp1, contains LSm and LSmAD domains similar to Atx2, an intrinsically disordered protein implicated in RNA biology and neurodegenerative disease [33]. The presence of the LSm domain in both human and yeast Ataxin-2 suggests that Atx2 can bind to RNA and regulate RNA metabolism and/or RNA processing [9,34]. The human *ATXN2* gene is located on chromosome 12q24 [35]. Initial studies showed that the Atx2 protein consists of 1313 amino acid residues, with a molecular weight of the mature peptide of approximately 140 kDa [36,37]. However, subsequent research utilizing reporter gene constructs has identified an alternative translation start codon, resulting in an Atx2 protein containing 1153 residues with a molecular weight of ~124 kDa [14,30,38,39]. It contains a polyglutamine (polyQ) tract of 23 glutamines at its N-terminus and includes an acidic region (residues 94–317). This region likely consists of two to seven exons and is hypothesized to contain two globular domains: LSm (residues 94–185) and LSmAD (residues 193–315) (see Figure 1A), which are the primary functional domains that potentially interact with RNA [38,40,41]. The LSmAD domain contains a clathrin-mediated trans-Golgi signal (^254^YDS) and an endoplasmic reticulum export signal (ERD) [24,42,43,44]. Dysfunction of the LSm and LSmAD domains has been associated with neurodegenerative diseases. The structural and functional domains of Atx2 exhibit distinct roles in neurodegenerative diseases, with the LSm domain potentially offering protection against neurodegeneration, while interactions with PAM2 and IDR both contribute to Atx2-induced cytotoxicity [10]. Therefore, the LSm and LSmAD domains of Atx2 are critical functional components, making them indispensable for its biological role from a structural biology perspective.

In general, members of the LSm protein family bind to RNAs through the LSm domain [45,46]. Previous studies have demonstrated that the LSm domain can enhance mRNA stability by interacting with the uridylate-rich element in the 3′-UTR of specific mRNAs, thereby increasing protein abundance [44]. The mature mRNA responds to cis-regulatory elements located in its 3′ untranslated region (UTR). 3′UTR cis elements mediate interactions with miRNAs and RBPs that govern mRNA fate [47,48] through abundant motifs such as AREs, AU-rich elements [49]. Atx2 regulates RNA metabolism and translation, and its mechanism remains largely unknown due to the lack of information on Atx2’s mRNA targets and how Atx2 interacts with RNA. Given the pivotal role of Atx2 in RNA processing and the pathogenesis of neurodegenerative disorders [44,50,51,52], we aim to elucidate the molecular mechanisms involved in its interaction with RNA. Such insights could facilitate the discovery of new therapeutic targets and enhance our understanding of the role of RBP in the complex regulatory network of gene expression [39]. This knowledge could contribute to the development of strategies aimed at modulating RBP function, which may benefit the treatment of associated diseases [27]. Neurodegenerative diseases, such as ALS and SCA2, involve the interaction between Atx2 and RNA. Understanding these molecular mechanisms can provide a theoretical foundation for comprehending the role of RBPs in gene expression regulation, as well as for developing treatment strategies and identifying new therapeutic targets. Consequently, we have designed and constructed a series of systematically shortened recombinants for LSm, LSmAD, LSm-LSmAD, and their mutants. These constructs will serve as a foundation for our investigation into the domain structure of Atx2 and the molecular mechanisms underlying its RNA binding.

In this report, the soluble recombinant LSmAD was purified using a two-step protocol involving His-tag affinity chromatography and size-exclusion chromatography, while the recombinant LSm protein was purified through the inclusion-body purification method. Additionally, we engineered several mutations in the LSm and linker regions of LSm-LSmAD to address the degradation issue. Furthermore, we utilized biophysical approaches to investigate the structural characteristics of the LSm and LSmAD domains from Atx2 and employed a chemical probing method to assess the sequence preference of LSm binding to RNA. Our findings indicate that LSmAD alone cannot fold well and does not bind to RNA, whereas LSm can bind RNA with high specificity.

## 2. Materials and Methods

### 2.1. Sample Preparation

#### 2.1.1. Sequence Analysis and Preparation of Expression Constructs

We first used *JPred* (https://www.compbio.dundee.ac.uk/jpred/) to predict the secondary structures of the LSm-LSmAD domain of Atx2. Based on secondary structures, we designed and constructed several recombinant plasmids. The expression vectors were derived from our own laboratory, including PET-22b, PH GB, PET-MG, and pp SUMO.

#### 2.1.2. Protein Expression and Purification

Soluble protein expression and purification: In a clean bench, add 1 µL of the recombinant plasmid to melted BL21 competent cells. Gently mix using a pipette and incubate on ice for 30 min. Heat shock in a 42 °C water bath for 90 s, then quickly transfer it to ice and allow it to stand for 2 min. Add 900 µL of LB liquid culture medium to the tube, position it at an angle in a 37 °C shaker, and incubate at 225 rpm for approximately 1 h. Centrifuge at low speed and discard the supernatant, leaving about 200 µL of the pellet. Resuspend the pellet and plate 100 µL onto the appropriate selective agar plate. Incubate overnight at 37 °C to isolate *E. coli* colonies containing the target protein expression gene. Inoculate a sterile shaking tube with 4 mL of LB medium containing the appropriate antibiotics, using a single colony from the overnight plate, and incubate overnight at 37 °C with shaking. Transfer the overnight culture to 1 L of pre-sterilized LB medium with antibiotics and grow in a 37 °C shaker until the OD600 reaches 0.6–0.8. Induce protein expression by adding IPTG to a final concentration of 0.2 mM, along with antibiotics, and incubate overnight at 22 °C. Centrifuge at 3500 rpm for 20 min to collect the cells, resuspend the pellet in 40 mL of lysis buffer containing PMSF, and lyse the cells using ultrasonic treatment for 30 min. Centrifuge at 13,000 rpm for 1 h, collect the supernatant, and purify the protein using a Ni-NTA affinity column (Roche, Switzerland). Wash with 20 mM imidazole and elute the target protein with 250 mM. Analyze protein expression and purification by SDS-PAGE [53]. Concentrate and further purify the protein samples using molecular sieve chromatography. For labeled samples, follow the same procedure but use M9 medium, with 15N-NH4Cl as the sole nitrogen source.

Inclusion bodies expression and purification [54,55]: The protein expression is comparable to that of soluble proteins. After overnight induction, the induced cell lysates were centrifuged at 13,000 rpm for 25 min, and the pellet was resuspended in binding buffer (50 mM Tris-HCl, pH 8.0, 100 mM NaCl, 1 mM EDTA, 1 mM PMSF). And then the cells were lysed by ultrasonic treatment for 30 min. The lysate was centrifuged to collect the pellet, which was washed twice by resuspension in wash buffer (50 mM Tris-HCl, pH 8.0, 100 mM NaCl, 1 mM EDTA, 1 mM PMSF, 1% Triton X-100) and centrifugation at 12,000 rpm for 10 min at 4 °C. The pellet was extracted with unfolding buffer (7.0 M urea, 50 mM Tris-HCl, pH 8.0, 0.5 M NaCl, 30 mM imidazole, 0.1% Triton X-100, 1 mM DTT, 1 mM PMSF), and the insoluble material was removed by centrifugation. The solubilized protein was applied to a 1 mL HisTrap FF column (Cytiva, Marlborough, MA, USA) equilibrated with buffer A (6.0 M urea, 50 mM Tris-HCl, pH 8.0, 0.5 M NaCl, 30 mM imidazole, 1 mM DTT, 1 mM PMSF). The column was washed with 30 column volumes of buffer A, and proteins were eluted with a step gradient of 0–100% buffer B (6.0 M urea, 50 mM Tris-HCl, pH 8.0, 0.5 M NaCl, 30 mM imidazole, 1 mM DTT, 1 mM PMSF, 500 mM imidazole). Protein refolding was performed by extensive dialysis against refolding buffer (50 mM Tris-HCl, pH 8.0, 2 M NaCl, 1 mM EDTA, 1 mM DTT). The final dialysis was carried out overnight.

#### 2.1.3. In Vitro Transcription and RNA Purification

The 3′-untranslated region (3′-UTR) of mRNA plays a significant role in regulating mRNA degradation, translation, and localization [56,57,58,59]. The 3′ UTR contains binding sites for RNA-binding proteins, such as AU-rich element binding proteins, which can affect the stability of the mRNA and thereby regulate its lifespan within the cell. AUUUA pentamers are commonly found in the U-rich regions of the 3′-UTR [60]. Cis-acting elements and trans-acting factors involved in RNA degradation that are located in the 3′ UTR include AU-rich elements [60,61], CA-rich elements [62], CU-rich elements, and GU-rich elements [63]. We designed RNA containing several elements in the 3′UTR. All RNA plasmids were synthesized by Sangon Biotech, Shanghai, China. The templates necessary for RNA synthesis were generated through PCR amplification. RNA was then produced using an in vitro transcription protocol as outlined by the laboratory. Following synthesis, the RNA was purified and separated using a polyacrylamide gel containing 8 M urea.

### 2.2. Size-Exclusion Chromatography

Size-exclusion chromatography (SEC) is a technique that employs porous gels with a specific pore size range as the stationary phase to separate mixture components based on molecular size. Freshly purified protein and nucleic acid samples, along with their complexes, were dissolved in tris buffer (25 mM Tris, 150 mM NaCl, pH 8.0) and subsequently applied to analytical columns Superdex 75 10/300GL (Cytiva, USA) and Superdex 200 10/300G (Cytiva, USA). The sample status was evaluated by analyzing the peak positions.

### 2.3. Circular Dichroism Spectroscopy

Circular dichroism spectroscopy (CD) experiments [64] were collected on a JASCO J 715 spectropolarimeter (JASCO, Tokyo, Japan). The sample concentration was 0.2 mg/mL, and the buffer was 20 mM PBS and 50 mM NaCl with a pH of 6.5. The cuvette light path was 0.1 cm and the grating width was 1 nm. The spectrum scanning range was 250 190 nm, the scanning speed was 10 nm/min, and the response time was set to 0.125 s. All samples were scanned 3 times, the average value was taken, and the collected data were denoised and smoothed. The final result was expressed in the average amino acid residue molar ellipticity unit deg cm^2^/dmol.

### 2.4. NMR HSQC Data Collection and Analysis

NMR (Nuclear Magnetic Resonance) data were acquired using Bruker 600 MHz and Agilent 800 MHz NMR spectrometers (Bruker, Berlin, Germany) at 25 °C, with sample concentrations ranging from 0.8 to 1 mM. The sample buffer for LSmAD was PBS. Data obtained from the NMR experiments were converted into a new format using NMR Pipe software (Version 9.9). In the two-dimensional ^1^H^15^N HSQC NMR spectrum, unfolded proteins show a marked reduction in signal dispersion in the proton dimension [65,66].

### 2.5. Electrophoretic Mobility Shift Assay (EMSA)

The protein sample was incubated with RNA in a binding buffer (10 mM Tris, pH 8.0; 25 mM KCl; 10 mM NaCl; 1 mM MgCl_2_; 10% glycerol; 0.5 mM DTT) for 20 min at 25 °C. The formation of the protein–RNA complex was confirmed using 1.5% TBE gel [53,67,68,69].

### 2.6. SAXS Data Collection and Analysis

All data were collected at the 19U2 beamline of the Shanghai Synchrotron Radiation Light Source. Samples were initially prepared and dissolved in SEC-FPLC buffer before data collection using SEC-SAXS. The sample volume was 100 µL and the flow rate was 0.5 mL/min, with images captured at a rate of 40 frames per minute. After data collection, the data were analyzed and processed using the small-angle scattering data processing software RAW. The process utilized a Kratky plot to assess the folding status of the samples [70,71].

### 2.7. SHAPE (Selective 2′-Hydroxyl Acylation Analyzed by Protection from Exoribonuclease) Probing Analysis

Prepare the protein and nucleic acid samples required for the experiment. Incubate these samples according to the EMSA protocol and then remove unreacted proteins through phenol–chloroform extraction. Next, aliquot 9 µL of the RNA samples (divided into sequencing and control groups) and 9 µL of the RNA–protein complex into separate tubes. Add 1 µL of DMSO and 1 µL of 1M7 to each tube, and incubate at 35 °C for 10 min. After incubation, add 500 µL of ethanol precipitation buffer and 1.5 µL of GlycoBlue to each sample. Precipitate the RNA by placing the samples in a −80 °C freezer for 40 min to 1 h. Following precipitation, centrifuge at 14,500 rpm at 4 °C for 45 min. Discard the supernatant, invert the tubes to allow the pellet to dry, and then resuspend the pellet in 9 µL of ultrapure water. Add 0.5 µL of FAM primer to each sample and perform reactions at 65 °C for 5 min and at 35 °C for an additional 5 min. After the reactions, lower the sample temperature to 4 °C and add 6 µL of RT mix (a mixture of reverse transcriptase buffer and dNTPs in a 5:1 ratio) to each sample, with additional ddA for the sequencing group. Incubate at 4 °C for 5 min and then raise the temperature to 49 °C and add reverse transcriptase for a 30-min reaction. Heat the samples to 95 °C, add 1 µL of 5 M NaOH, and incubate for 5 min. Add acid stop dye and heat at 95 °C for an additional 5 min to terminate the reaction [72]. Send the samples to Sangon Biotech for sequencing and analyze the results using ShapeFinder (Version 1.0) [73,74,75].

## 3. Results

### 3.1. Expression and Purification of Recombinant Proteins

In the initial phase of our study, secondary structure prediction was employed to design and construct several recombinant plasmids encoding LSm, LSmAD, LSm-LsmAD, and various mutants of LSm-LSmAD (Figure 1A). The biggest problem encountered when purifying LSm is its insolubility. In order to obtain the LSm protein, we tried to apply GB1 and SUMO fusion tags for expression and purification (Figure 1A), but the situation did not improve. Next, we tried ammonium sulfate precipitation, but it had very little effect. Finally, we opted for inclusion bodies purification, which can obtain a reasonable amount of LSm protein samples through optimization The LSm (residues 82–184) was cloned into pET-22b (+) vector flanked by the NdeI and XhoI restriction sites and the recombinant protein was obtained in the insoluble fraction, and then the inclusion bodies of LSm were purified (Figure S1A), as detailed in the Materials and Methods section. Briefly, the pellet was dissolved in the denaturing buffer and the extract was centrifuged to remove any insoluble materials, and then the supernatant was applied to a Histrap FF column for further purification. The protein band appeared to be between 10~15 kDa, corresponding to its theoretical molecular weight of 11.4 kDa. Next, we followed with the standard protocols to purify LSmAD, which can obtain a purified sample of LSmAD (Figure 1B and Figure S1B). The band in ~18 kDa indicated the soluble protein of LSmAD, consisting of its theoretical molecular weight of 15 kDa.

During the purification process of LSm-LSmAD, we consistently faced problems of protein degradation and aggregation. To address these issues, we engineered a series of mutations in the LSm and linker regions of LSm-LSmAD, including M1 (D184N + S185Q), M2 (S155Q + S157T), and M3 (S155Q + S157T + D184N + S185Q) (Figure 1A). We found that the M2 mutation could effectively overcome the degradation problem; hence, it was adopted in subsequent experiments (Figure S1C). Its molecular weight appeared to be around 30 kDa, close to the theoretical value of 28 kDa. We then focused on preparing both the wild-type LSm and the S155Q + S157T mutant of LSm-LSmAD for our subsequent experiments (Figure 1C). The successful preparation of these proteins laid the foundation for our detailed structural and functional analyses.

### 3.2. The Flexible Structure of LSmAD Lacks RNA Binding

**Figure 2:**
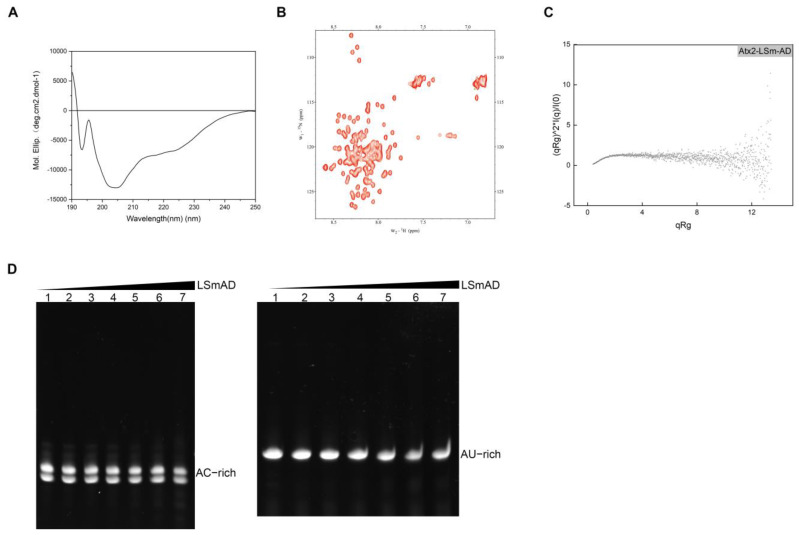
Structural and functional analysis of LSmAD. (**A**) CD spectrum of LSmAD. (**B**) HSQC spectrum of LSmAD. (**C**) Kratky plot of LSmAD. (**D**) EMSA for characterizing the interaction of LSmAD with AC-rich and AU-rich RNA. Left, EMSA for characterizing the interaction of LSmAD with AC-rich RNA; right, EMSA for characterizing the interaction of LSmAD with AU-rich RNA. The top black graph illustrates the gradual increase in protein dose. Lanes 1–7 represent the molar protein/RNA molar ratio of 0, 0.25, 0.5, 1, 2, 4, and 6, respectively.

To acquire structural information about the LSmAD domain, we began characterizing its secondary structures by using circular dichroism spectroscopy (CD). The resulting CD spectrum exhibited a distinct negative peak at 202 nm, accompanied by a subtle shoulder at 220 nm (Figure 2A), indicating the modest presence of α-helical structural elements. Subsequent analysis using ^1^H-^15^N NMR spectroscopy (HSQC) showed that most aminde signals clustered together in the 8.1–8.5 ppm range (Figure 2B), resembling the reduced signal dispersion from unfolded proteins. These spectroscopic data suggest that LSmAD may intrinsically exist in multiple conformations in solution. To further elucidate the overall structural characteristics of LSmAD, we conducted small-angle X-ray scattering (SAXS) experiments. Kratky curve analysis of the SAXS data demonstrated that LSmAD formed a flexible structure (Figure 2C). Collectively, the CD, NMR, and SAXS analyses indicate that LSmAD exhibits a notable uniformity and is devoid of aggregation while possessing a considerable degree of flexibility. The LSmAD domain may lack a homogeneous tertiary structure, suggesting a propensity for multiple conformations.

Given the inferred flexibility of the LSmAD structure, we next sought to determine its potential to directly interact with RNA in vitro. We designed several RNA sequences enriched with AU and AC, namely AU-rich and AC-rich sequences, respectively (Table 1). These RNAs were prepared by in vitro transcription (IVT) and purified via gel extraction. We applied EMSA to identify the RNAs bound to LSmAD. As shown in Figure 2D, the AC-rich RNA exhibited two bands in the basal region, indicating that it may exist in two conformations in the solution, while the AU-rich RNA displayed a single band. Subsequently, we tested whether AC-rich and AU-rich RNA could bind to LSmAD by gradually increasing the protein concentrations. The data showed that, with the increasing dosage of LSmAD, the basal bands of both AC-rich and AU-rich RNA did not change or shift (Figure 2D), indicating that LSmAD did not interact with either the AU-rich or AC-rich RNA. Therefore, we conclude that LSmAD alone is incapable of binding RNA.

### 3.3. LSmAD Assists LSm Folding and Facilitates Its Binding with AU-Rich RNA

**Figure 3:**
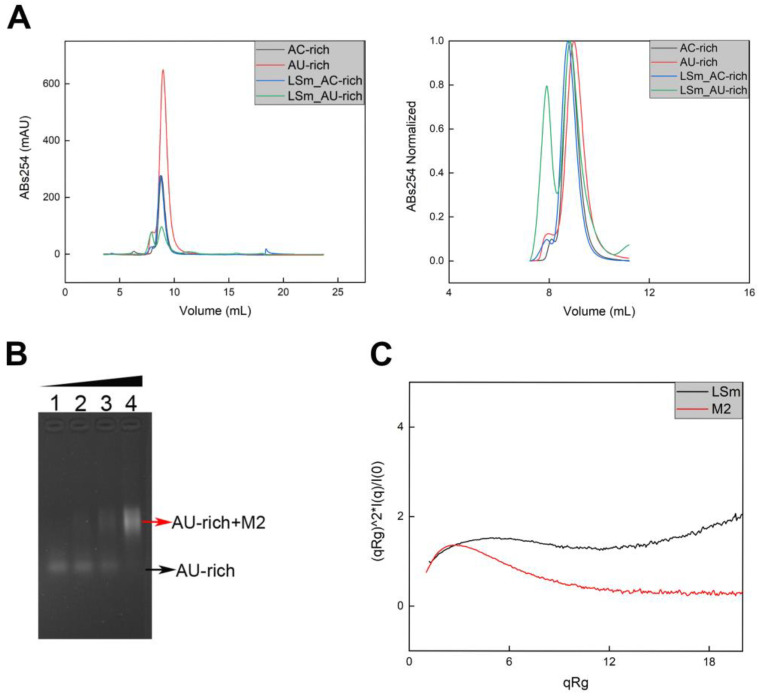
(**A**) SEC-FPLC analysis of the interaction of LSm with AU-rich and AC-rich RNA sequences, respectively. The right graph shows the normalized curves. (**B**) EMSA analysis of M2 with the AU-rich RNA sequence. The numbers 1–4 represent the M2 to RNA molar ratios of 0, 5, 10, and 20. (**C**) Kratky plot of LSm (black) and M2 (red).

Since the LSmAD domain alone does not bind RNA, we turned to LSm for our next investigation. Firstly, we used SEC-FPLC to determine whether LSm binds to RNA. As shown in the Figure 3A, the peak position of the mixture of LSm and AC-rich RNA remained almost unchanged compared to that of AC-rich RNA alone. In contrast, an additional peak appeared ahead of the AU-rich peak in the mixture sample of LSm and AU-rich RNA, indicating that LSm specifically binds to the AU-rich sequence but not to the AC-rich sequence.

To investigate the impact of LSmAD on LSm binding to RNA sequences, we utilized EMSA to determine whether the M2 mutant of LSm-LSmAD binds to AU-rich RNA. The result indicated that M2 binds to AU-rich sequences with a moderate affinity (Figure 3B). As the molar ratio of M2 to RNA increases, the intensity of free RNA (lower band) decreases gradually, while that of the M2-RNA complex (upper band) increases significantly. At the molar ratio to 20, the free RNA disappears entirely in the gel and the intensity of the complex reaches saturation. These data suggest that M2 specifically binds to AU-rich RNA with a stoichiometric manner.

To elucidate the conformational landscape of the complex formed by LSm-LSmAD and AU-rich RNA, we utilized SAXS to assess the structural attributes of LSm and M2 independently. Kratky plot analysis revealed that M2 exhibited a more ordered folding pattern compared to LSm alone (Figure 3C), implying that the LSmAD domain plays a role in facilitating the proper folding of the LSm domain. This observation suggests that the LSmAD domain may modulate the structural integrity of the LSm domain.

Collectively, in Atx2, the LSm domain, rather than LSmAD, binds to AU-rich RNA, while LSmAD facilitates the folding of LSm and enhances the binding affinity of the LSm moiety for AU-rich RNA.

### 3.4. ATX2 Preferentially Binds to U-Rich Sequences but Not to CA-Rich Sequences

Having established that the LSm domain of Atx2 specifically binds to the AU-rich sequences, we next aimed to determine whether Atx2 also interacts with other elements within the mRNA 3′-UTR. We designed three additional RNA constructs, namely RE1, RE2, and RE5 (Table 2), with the AU-, CU-, GU- and CA-rich sequences flanked by a standard SHAPE process. Subsequently, we performed a SHAPE assay to footprint Atx2 on RE1, RE2, and RE5, respectively.

**Figure 4:**
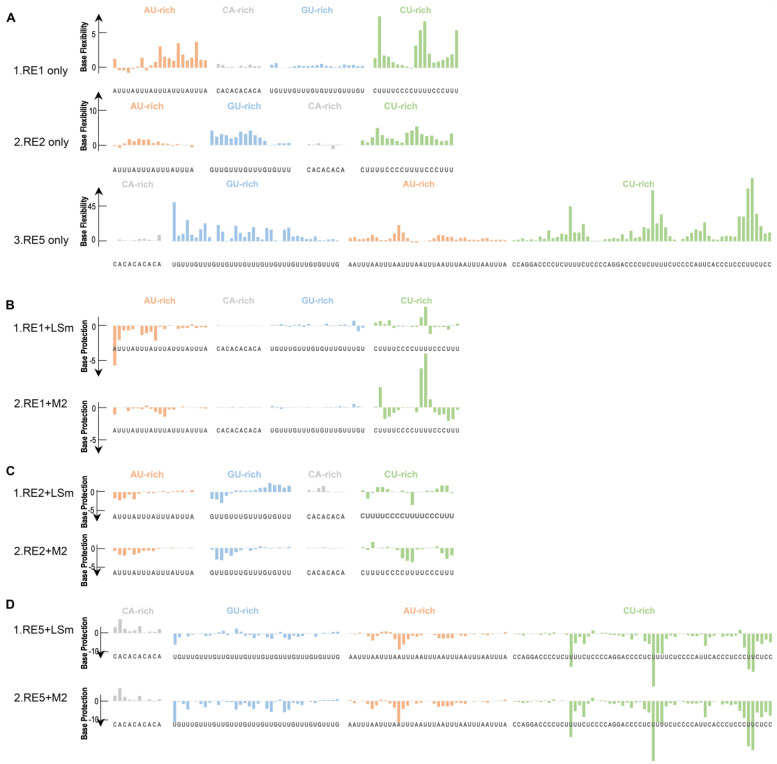
The LSm domain of Atx2 recognizes the U-rich sequences. The color bars represent reduced SHAPE reactivity. The residues are indicated on the *X*-axis, while the CA-rich, GU-rich, AU-rich, and CU-rich sequences of RNA are labeled in grey, blue, orange, and green, respectively. The height of the bar graph indicates signal strength. Colored upper bars of RNA only represent reduced SHAPE reactivity. Downward bars in the RNA + protein row indicate the degree of base protection after the addition of protein. (**A**) Control for SHAPE analysis. (**B**) SHAPE analysis was carried out for RE1: LSm binding to RE1 (row1) and M2 binding to RE1 (row2). (**C**) SHAPE analysis was executed for RE2: LSm binding to RE2 (row1) and M2 binding to RE2 (row2). (**D**) SHAPE analysis was performed for RE5: LSm binding to RE5 (row1) and M2 binding to RE5 (row2).

We assessed the impact of the Atx2 protein on RNA sequences by examining both protein-bound and protein-free states. Upward bars of RNA only represent base flexibility, while downward bars of RNA–protein indicate the degree of protection after binding the LSm/M2 protein. The RNA-only controls are presented in Figure 4A, where rows 1, 2, and 3 correspond to RE1, RE2, and RE5. Figure 4B illustrates the binding properties of RE1 in the presence of either the LSm or M2 protein. The SHAPE reactivity values for the CA-rich (gray bar) and GU-rich (blue) sequences remained stable in both the protein-free and protein-bound states. In contrast, the AU-rich (orange bar) and CU-rich (green bar) sequences exhibited higher SHAPE reactivities in the protein-free state, indicating higher conformational dynamics in the absence of protein. When comparing the SHAPE reactivity upon the addition of LSm (Row 1) and M2 (Row 2), both AU-rich and GU-rich sequences demonstrated protection. Specifically, the AU-rich sequences showed a higher level of protection upon the addition of LSm compared to M2. Conversely, for GU-rich sequences, M2 induced greater protection than LSm. These findings indicate that the M2 mutation influences LSm binding preferences for RNA sequences.

To further explore the relationship between protein binding and RNA structure, we designed an additional RNA (RE2) by rearranging the four sequences (Table 2). The SHAPE reactivity of the CA-rich regions (gray bar) remained unchanged regardless of the presence of protein, while the reactivity of the AU-rich (orange bar) and CU-rich (green bar) sequences exhibited reduced SHAPE reactivity (Figure 4C). RNA sequences enriched in AU motifs were found to be protected by both LSm and M2 proteins. Comparative analysis revealed that M2 provided a more substantial protective effect on AU-enriched sequences than LSm, suggesting a potentially higher affinity of M2 for these motifs. This enhanced protective effect of M2 may indicate a superior binding affinity for AU-rich sequences. Moreover, for RNA sequences rich in GU and CU, protective effects from both proteins were observed, with M2 demonstrating a more pronounced protective effect than LSm. These findings collectively suggest that M2 possesses a stronger binding capacity for the U-rich sequences. The LSmAD domain enhances the affinity of LSm for U-rich sequences, suggesting that LSmAD may assist in modulating the interaction between LSm and RNA during RNA processing and regulation.

Finally, we designed RE5 to further optimize the RNA sequence (Table 2). The SHAPE reactivity of the CA-rich sequences remained almost unchanged regardless of the presence of protein, whereas the reactivity of the AU-rich and CU-rich sequences exhibited notable alterations (Figure 4D). Upon the addition of LSm or M2 proteins, the SHAPE reactivities of GU-, AU-, and CU-enriched RNA sequences were diminished in a comparable manner, with the most pronounced effects observed in the CU-enriched sequences. Overall, for the RE5 RNA, LSm and M2 cannot bind to the CA-rich sequences but can bind to the AU-rich, GU-rich and CU-rich sequences, while LSmAD promotes LSm binding to U-rich sequences.

## 4. Discussion

Atx2 is associated with a range of human diseases, including Parkinson’s disease (PD), spinocerebellar ataxia (SCA1, SCA2, MJD), amyotrophic lateral sclerosis (ALS), primary open-angle glaucoma (POAG), and obesity/type I diabetes [14]. As an evolutionarily conserved protein found across eukaryotes, Atx2 contains the N-terminal LSm and LSmAD domains, which exhibit a high degree of conservation among various species. The LSm domain is involved in RNA binding, while the LSmAD domain contains a crucial signal for trafficking from the endoplasmic reticulum to the extra-Golgi apparatus [44]. These functions are evolutionarily conserved, highlighting the importance of these domains in RNA metabolism across diverse species [9,29,41].

Atx2, a member of the LSm family, tends to form 6-, 7-, or 14-mers [76]. Our experimental observations indicate that both LSm and LSm-LSmAD variants of Atx2 tend to aggregate, consistent with the propensity for LSm proteins to engage in multimeric complexes. LSm domain proteins are involved in a plethora of RNA processing events, including RNA modification, pre-mRNA splicing, and mRNA decapping and degradation [77,78]. We used CD spectroscopy to study the secondary structure of LSmAD instead of LSm because the LSm family usually contains a conserved Sm domain, composed of 5–7 antiparallel β-strands (β-sheet), forming a typical β-barrel structure [76,79,80]. CD spectroscopy often fails to provide acceptable results on α/β-mixed or β-structure–rich proteins. The issue arises from the spectral diversity of β-structures, which has been considered an intrinsic limitation of the technique [81].Therefore, we believe that CD spectroscopy is not suitable for studying the secondary structure of the LSm construct used in this study. The LSm domain of Atx2 is likely crucial for its interaction with RNA, although no specific RNA has been identified that directly binds to Atx2 [9]. In this study, we employed EMSA and SEC-FPLC to investigate the interaction between LSm, LSmAD, and LSm-LSmAD with RNA. Our findings reveal that both LSm and LSm-LSmAD can bind RNA, whereas LSmAD alone cannot, confirming that the RNA-binding region in Atx2 resides in the LSm domain rather than in the LSmAD domain. For the EMSA experiment, we followed the published methods [8,68,69,82,83]. As shown in Figure 3B, M2 can bind to AU-rich sequences, and the amount of M2-RNA complex increases with an increase in the molar ratio of protein to RNA, reaching saturation at a molar ratio of 20. However, no matter how it is optimized, the LSm-AU-rich complex band always migrates very slowly, and the EMSA of LSm and AU-rich sequences cannot reach saturation, as shown in the Figure S3. Therefore, we accepted this consistent result and decided to remove the EMSA experiment section about related to LSm and AU-rich sequences, and turned to explaining its physiological importance. Atx2 may form oligomers through the LSm domain [84,85,86]. The formation of oligomers may be the main reason for the slow migration and inability to saturate the LSm-RNA complex. Atx2 aggregation appears to drive the pathology of neurodegenerative diseases [85,86]. Furthermore, Atx2 serves as a component protein of stress granules (SGs), significantly influencing their assembly and regulation [10,32,44].

Previous studies have demonstrated that the LSm domain can enhance mRNA stability by interacting with the uridylate-rich element in the 3′-UTR of specific mRNAs, thereby increasing protein abundance [44]. Additionally, the LSm domain is believed to play a role in translational regulation, particularly in the activation and repression of mRNA translation [10]. The LSmAD domain, which contains the ER export signal and is essential for protein cleavage from the ER, Golgi apparatus, or plasma membrane, suggests that it plays a pivotal role in the subcellular localization and trafficking of Atx2 [44].

Our investigation into the solution structure has revealed that LSmAD is inherently flexible. This flexibility indicates dynamic structural plasticity, which may influence its functional interactions with other biomolecules. Further SAXS analysis of LSm and LSm-LSmAD in solution showed that LSm-LSmAD exhibits a higher degree of folding compared to LSm alone, indicating that the LSmAD domain may facilitate the structural folding of LSm. The LSm and LSmAD domains of Atx2 are essential to its function.

One prominent cis-regulatory region is the AU-rich element (ARE) [8,60,87,88]. ARE, characterized by its sequence rich in adenine (A) and uracil (U) nucleotides, is known to target mRNA for rapid degradation [88]. The interaction of Atx2 with ARE in 3′-UTR may modulate the stability of many target mRNAs [89]. Atx2 can either enhance or suppress the translation of specific mRNAs [89,90]. It is involved in various processes, such as translation activation, translation repression, mRNA stability, and mRNP particle assembly [10]. Although it has been proposed that Atx2 plays a role in the regulation of RNA metabolism and translation, the underlying mechanisms remain largely unknown, due to a lack of information regarding the mRNA targets of Atx2, let alone how Atx2 interacts with RNA. Our results revealed the specific domain of Atx2 and its sequence preferences.

Research has shown that multiple RBPs regulate mRNA stability through binding to AREs [88]. Our study revealed that the LSm domain can specifically bind to AU-rich but not AC-rich sequences, coinciding with the previous observation that Atx2 recognizes AU-rich elements as binding motifs [8]. Furthermore, we demonstrated that Atx2 can bind to GU-rich and CU-rich sequences, but not to the CA-rich sequence.

Our findings provide compelling evidence that the LSm domain within Atx2 serves as the primary RNA-binding motif, with the LSmAD domain exerting a positive regulatory influence on the interaction of Atx2 with RNA. This discovery emphasizes the significance of LSm’s ability to selectively engage U-rich sequences, exhibiting specificity not only for the RNA sequence but also for its tertiary structure, which is important for elucidating the binding mechanism of Atx2 to RNA. The presence of U-rich sequences is essential for the precise control of gene expression, particularly in the rapid response of cells to changes in the internal and external environment [91]. In particular, AU-rich elements (AREs) play a central role in regulating mRNA stability [88]. Atx2 can specifically bind to AU-rich sequences and may play a central role in mRNA stability. As a member of the RBPs, Atx2 may significantly modulate mRNA stability and translation. In addition, the dysregulation of ARE-containing mRNAs has been correlated with pathological states such as cancer, chronic inflammation, and autoimmune diseases, suggesting a link between the regulatory mechanisms of U-rich sequences and disease pathogenesis [88].

## 5. Conclusions

In this study, the recombinant LSmAD was successfully expressed in soluble form and subsequently purified using a two-step protocol, while recombinant LSm was purified from inclusion bodies. Moreover, to address the degradation issue encountered in the LSm-LSmAD domain, our strategic approach to protein engineering, specifically the introduction of the M2 mutation, thereby enabled a more in-depth exploration of the structural and functional properties of the LSm-LSmAD sample. The RNA-binding domain of Atx2 is the LSm domain. The LSmAD by itself is unable to bind RNA; however, it can enhance the RNA-binding activity of LSm and facilitate its folding.Atx2 can specifically bind to U-rich sequences. The multifaceted role of U-rich sequences in post-transcriptional regulation is not only central to the maintenance of gene expression homeostasis but also potentially pivotal in disease etiology and progression. The LSm and LSmAD domains in Atx2 play a key role in RNA metabolism and translation regulation and may be related to the development of a variety of diseases. Further studies may reveal how these domains are involved in the development of diseases and their potential as therapeutic targets. These findings provide new perspectives for future therapeutic strategies targeting the regulatory mechanisms of U-rich sequences.

## Figures and Tables

**Table 1:** The sequences of AU-rich and AC-rich RNA.

RNA Name	Sequence
AU-rich	UAAUACGACUCACUAUAGGCCUUCGGGCCAAAUUU UUAUUUUUAUUUUUAUUUUUAUUUUUUCGAUCCGG UUCGCCGGAUCCAAAUCGGGCUUCGGUCCGGUUC
AC-rich	UAAUACGACUCACUAUAGGCCUUCGGGCCAAACCC CCACCCCCACCCCCACCCCCACCCCCUCGAUCCGG UUCGCCGGAUCCAAAUCGGGCUUCGGUCCGGUUC

**Table 2:** RNA sequences resulting from SHAPE analysis.

RNA Name	Sequence
RE1	UAAUACGACUCACUAUAGGCCUUCGGGCCAAAUUUAUUUAUUUAUUUA UUUAGCUGACGAUCCACACACACAGGAAUCGACUCUGUUUGUUUGUGU UUGUUUGUACUGAAUUGGCACUUUUCCCCUUUUCCCUUUCUGGACUGG CAUCGAUCCGGUUCGCCGGAUCCAAAUCGGGCUUCGGUCCGGUUC
RE2	UAAUACGACUCACUAUAGGCCUUCGGGCCAAAUUUAUUUAUUUAUUUA GCGAGAAGUUGUUUGUUUGUGUUUGACGUCUGUGUGGACGUCACACAC AGGAUGCAUCGGACCUUUUCCCCUUUUCCCUUUUCGAUCCGGUUCGCCG GAUCCAAAUCGGGCUUCGGUCCGGUUC
RE5	UAAUACGACUCACUAUAGGCCUUCGGGCCAAACCGUACACACACACAUG GCUAGACGGUAUGUUUGUUUGUUGUUUGUUUGUUGUUUGUUUGUGUUU GACGUAGGAAUUUAAUUUAAUUUAAUUUAAUUUAAUUUAAUUUACAGG CUACGUAGGCCAGGACCCCUCUUUUCUCCCCAGGACCCCUCUUUUCUCC CCAUUCACCCUCCCUUCUCCAGAGCGAUUCGAUCCGGUUCGCCGGAUCC AAAUCGGGCUUCGGUCCGGUUC

## Data Availability

The original contributions presented in this work are included in this article; further inquiries can be directed to the corresponding author.

## Supplementary Materials

The following supporting information can be downloaded at: https://www.mdpi.com/article/10.3390/cells14050383/s1, Figure S1. Purification of the recombinant domains of Atx2. Figure S2. Purification of AC-rich RNA. Figure S3. EMSA for characterizing the interaction of LSm with AU-rich RNA under different conditions.

## Abbreviations

Atx2	Ataxin-2
LSm	Sm-like
LSmAD	LSm-associated domain
RBD	RNA-binding domain
EMSA	Electrophoretic mobility shift assay
SHAPE	Selective 2′-hydroxyl acylation analyzed by protection from exoribonuclease
SAXS	Small-angle X-ray scattering
NMR	Nuclear magnetic resonance
PAM2	Poly(A)-binding protein-interacting motif
IDRs	Intrinsically disordered regions
SCA2	Spinocerebellar ataxia type II
ALS	Amyotrophic lateral sclerosis
CD	Circular dichroism spectroscopy
IVT	In vitro transcription
SEC	Size-exclusion chromatography
