# Supplementary material for: The LSmAD Domain of Ataxin-2 Modulates the Structure and RNA Binding of Its Preceding LSm Domain
Source: Cells. 2025 Mar 6;14(5):383. doi: 10.3390/cells14050383 (PMC11898529; doi:10.3390/cells14050383)
Supplement: Supplementary file 1 [file cells-14-00383-s001.zip › cells-3430325-supplementary.pdf]

Supplementary information

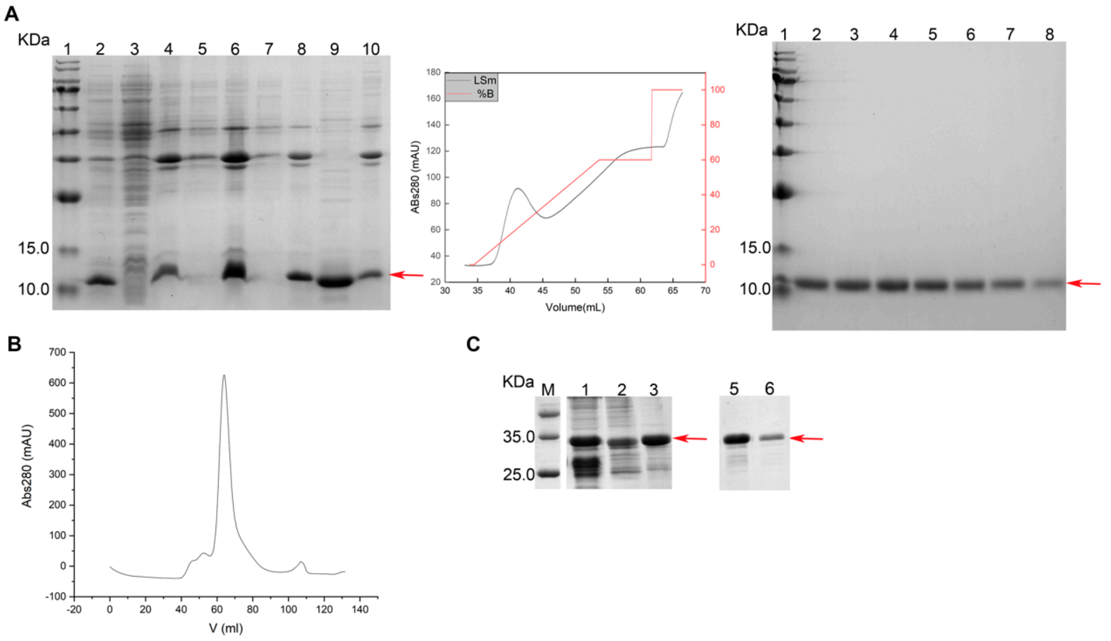

Figure S1 Purification of the recombinant domains of Atx2. (A) Left: lane 1, molecular weight marker; lane 2, cell lysates (induced); lane 3, supernatant-1; lane 4, precipitate-1; lane 5, supernatant-2; lane 6, precipitate-2; lane 7, supernatant-3; lane 8, precipitate-3; lane 9, supernatant-4; lane 10, precipitate-4. The middle graph shows the purification profile of LSm through HistrapFF column. Right: the peak fraction of LSm from His-tag affinity purification; lane 1, molecular weight marker; lane 2-8, peak frqaction from the middle graph. (B) SEC-FPLC purification of LSmAD. (C) Purification of LSm-LSmAD and its mutants. The left panel shows the differences among WT and its mutants. M, protein marker; lane 1, WT; lane 2, M1; lane 3, M2. The right illustrates the differences between the M2 and M3 mutants. lane 5, M2; lane 6, M3.

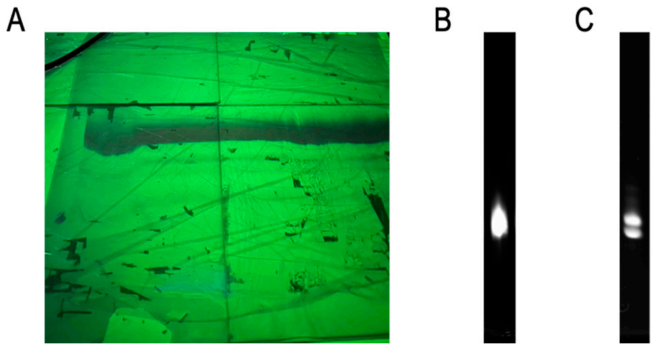

Figure S2 Purification of AC-rich RNA**.** (A) *In vitro* transcription result of AC-rich RNA. (B) The purified RNA after *in vitro* transcription was detected by polyacrylamide gel. (C) Native gel assay of the AC-rich RNA following renaturation.

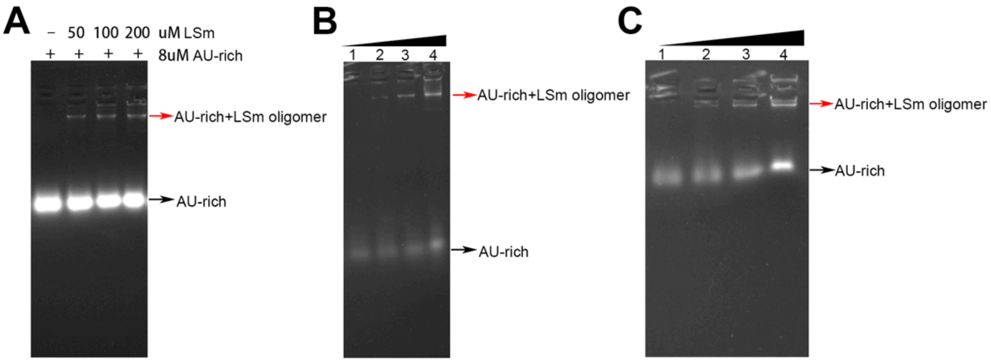

Figure S3 EMSA for characterizing the interaction of LSm with AU-rich RNA under different conditions. (A) Excessive amounts of protein and RNA. (B) The concentration of AU-rich RNA was 0.8 μM, while the molar ratio of LSm to AU-rich RNA was 0, 5, 10, and 20. Binding buffer: 10 mM Tris (pH 8.0), 25 mM KCl, 10 mM NaCl, 1 mM $MgCl_2$, 10% glycerol, 0.5 mM DTT. (C) The concentration of AU-rich RNA was 0.8 μM, while the molar ratio of LSm to AU-rich RNA was 0, 5, 10, and 20. Binding buffer: 10 mM Tris (pH 8.0), 25 mM KCl, 10 mM NaCl, 1 mM $MgCl_2$, 10% glycerol, 0.5 mM DTT, and 0.5 mg/mL BSA.
